# Cost-effectiveness of a statewide public health intervention to reduce cardiovascular disease risk

**DOI:** 10.1186/s12889-019-7573-8

**Published:** 2019-09-06

**Authors:** Lauren Smith, Adam Atherly, Jon Campbell, Nick Flattery, Stephanie Coronel, Mori Krantz

**Affiliations:** 1 School of Public Health, University of Colorado, Aurora, USA; 2 Center for Health Services Research, Larner College of Medicine, University of Vermont, 89 Beaumont Ave, Burlington, VT 05405 USA; 3 School of Pharmacy University of Colorado, Aurora, USA; 4 Colorado Prevention Center, Denver, USA; 5 Denver Health Medical Center Cardiology Division, Denver, USA; 6 School of Medicine, University of Colorado, Aurora, USA

## Abstract

**Background:**

The cost-effectiveness of community health worker (CHW)-based cardiovascular disease (CVD) risk-reduction interventions is not well established. Colorado Heart Healthy Solutions is a CHW-based intervention designed to reduce modifiable CVD risk factors. This program has previously demonstrated success, but the cost-effectiveness is unknown. CHW-based interventions are potentially attractive complements to healthcare delivery because laypersons implement the intervention at a lower cost relative to medical care and may be attractive in rural settings with limited clinical resources.

**Methods:**

CHWs performed screenings and provided ongoing participant support within predominantly rural communities. A point-of-service software tool was used to generate 10-year Framingham CVD risk scores and assist CHWs to make medical referrals and provide ongoing individualized support for lifestyle changes. A sample of program participants returned for reassessment of risk factors. We calculated quality-adjusted life years (QALYs) gained and program costs using a Markov model. Transition probabilities were calculated using Framingham risk equations or derived from the literature using the observed mean reduction in 10-year CVD risk score over of 37- months follow-up. Program cost-effectiveness was calculated for both at-risk (abnormal baseline CVD risk factors) and overall program populations.

**Results:**

The base-case scenario evaluating a 52-year-old male participant revealed an incremental cost savings of $3576 and a gain of 0.16 QALYs associated with the intervention. Cost savings were greater in at-risk populations. The economic dominance of the model was robust in multiple sensitivity analyses.

**Conclusions:**

A community-based CVD intervention demonstrated to reduce CVD risk is cost-effective. This suggests that population-based public health programs may have the potential to complement primary care preventative services to improve health and reduce the burden of traditional medical care.

## Background

Cardiovascular disease (CVD) is the leading cause of morbidity and mortality in the United States, with approximately 1 in 3 deaths and 15% of U.S. health expenditures in 2011 attributed to CVD [1]. Much of the incidence is attributable to modifiable lifestyle risk factors, with one study estimating that potentially modifiable risk factors account for over 90% of population attributable risk of myocardial infarction [2]. However, interventions addressing lifestyle risk factors, such as promotion of smoking cessation, physical activity programs, and targeting dietary changes, have shown limited effectiveness [3, 4]. A systematic review found mixed effectiveness and small effect sizes in such interventions, though the impact would be potentially substantial at larger population levels [4].

One approach to improving lifestyle and CVD risk factors is the use of community health workers, in which laypersons are trained to implement disease-specific health coaching interventions. Evidence of the effectiveness of community health worker (CHW)-based interventions has been mixed [5–7]. A recent systematic review, however, found community health worker-based interventions to be effective in improving health among vulnerable individuals with chronic disease [8]. Of the 26 studies reviewed that targeted cardiovascular disease, 60% were found to decrease risk factor burden. Information on the cost-effectiveness of CHW-based interventions remains limited, particularly among studies focused on CVD risk reduction. The key effects in successful studies were improvements in lipid profile, blood pressure, hemoglobin A1C and global CVD risk. Despite the dearth of cost-effectiveness studies, CHW-based interventions may augment healthcare delivery by providing ongoing support outside of the confines of the clinic. This may be particularly important in rural areas where geographic and financial barriers limit ongoing preventive care. There is some evidence that a community health worker-based intervention is cost-effective in controlling diabetes [9].

Colorado Heart Healthy Solutions, a CHW-based intervention was previously demonstrated to reduce global cardiovascular disease risk among vulnerable individuals [10]. Although there is an extensive literature regarding the cost effectiveness of healthcare interventions, less is known about the cost effectiveness of population health programs. Because hospitals and accountable care organizations are beginning to accept financial risk for the health of large populations of patients, there is new momentum for the development of public health-clinical care delivery models that aim to reduce preventable illness [11]. Given this background, we sought to determine the cost-effectiveness of Colorado Heart Healthy Solutions in reducing CVD burden by assessing program costs and projected reductions in CVD events.

## Methods

### Intervention and sample

Recruitment sites include churches, local businesses, homeless shelters, and local public health clinics. Health screenings are performed on-site, and include blood pressure, weight, height, and point-of-service cholesterol and diabetes screenings (Cholestech, Inverness Medical, Hayward CA) [10]. This information, combined with targeted CVD health history, access to care, diet and physical activity data, is input into a central data support module, the Outreach Screening and Referral (OSCAR) system. OSCAR is a screening and decision support tool (CPC Community Health, Aurora CO) used to generate 10-year CVD risk scores and provide cues for appropriate healthcare referrals, incorporating national guidelines based upon participant’s risk factors. The OSCAR system is tablet based and synchronizes to a master database using a web server to provide access to screening results and reporting. CHWs create action plans with individual participants and based upon CVD risk, initiate medical referrals, provide smoking cessation aids, and navigate interested individuals into nutritional and exercise programs. Subsequently, CHWs schedule follow-up calls for ongoing participant support to ensure follow-through with health-promotion action plans. Participants were reminded to return, > 3 months following the initial screening for retesting.

A total of 698 individuals received the intervention. Colorado Heart Healthy Solutions led to a 0.8% reduction Framingham Risk Score among the overall population and a 2.0% Framingham Risk Score reduction among at-risk individuals, defined as those participants with elevated baseline risk factor values upon initial screening [10].

### Analysis

**Fig. 1:**
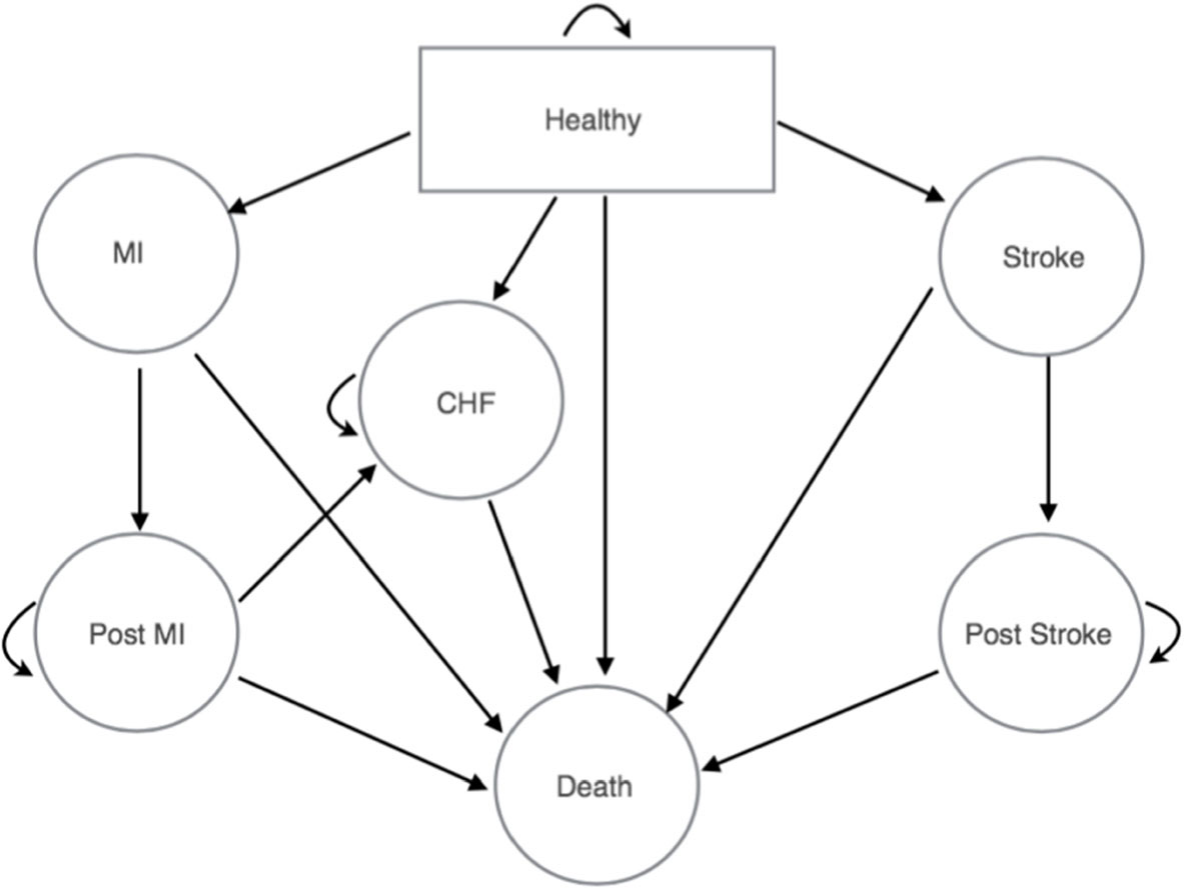
Markov model. All patients start in the healthy state and can transition to myocardial infarction (MI), stroke, or congestive heart failure (CHF) states. Subsequent transitions are indicated by arrows. Model cycles on a one-year timeframe

A Markov model was constructed to calculate costs and outcomes. We used a cost-utility analysis, comparing quality-adjusted life years (QALYs) gained to the net costs. The Markov model includes seven mutually exclusive states: normal health, acute myocardial infarction (MI), post-MI, stroke (ischemic and hemorrhagic), post-stroke, congestive heart failure (CHF), and death (Fig. 1). All participants begin in normal health state, and then move through the model based on transition probabilities calculated from their risk factors. If an acute event (MI or stroke) occurs, the subject can move either to a post-event state or death. Subjects cannot return to a healthy state following an adverse event. Because MI is the leading cause of CHF in the US [1], a subject can also move from the healthy and post-MI states to the CHF state. If the individual moves to the CHF state, they remain in this state until death. Additionally, subjects can move from the normal health state directly to death, due to non-cardiovascular related mortality. The cycle length is 1 year, and the time horizon is 30 years. The comparison to the Colorado Heart Healthy Solutions intervention was to those not receiving the intervention, which assumes that individuals receive standard medical care and progress between health states based on the probabilities given in the Framingham study (described below).

### Model inputs: transition probabilities

Transition probabilities were calculated using risk estimates based on the Framingham Heart Study, which were converted to one-year event probabilities [12, 13]. The model’s risk factors were populated from Colorado Heart Healthy Solutions participants screened between February 2010 and June 2015. Only participants who had full test/retest values available were included (n = 698). We defined at-risk participants as any individual with an uncontrolled risk factor or having a Framingham Risk Score of 10% or greater.

Risk factors used to calculate the transition probabilities into and out of the health states described previously include age, sex, systolic blood pressure, total cholesterol, high-density lipoprotein cholesterol, diabetes status and smoking status. Left ventricular hypertrophy and valvular heart disease status were not available in the dataset, so participants were assumed to be without these diseases in the main analysis. Transition probability calculations based on the Framingham Heart Study were used [18]. Recurrent CVD events are not used because these events in those with incident (new) CVD are relatively low in contemporary practice.

All risk factors were held constant, other than age, which increased yearly. Transition probabilities from the healthy state to each of the adverse outcome states were calculated for each year of age, as aging substantially modifies global CVD risk. An age-specific calibration factor was subsequently applied to bring the model’s incidence rates in line with expected rates. The calibration factor was determined by age group, using published incidence rates for each CVD event outcomes [14]. The published incidence rates were divided by the observed incidence rates calculated by the model. This number was rounded down to the nearest whole number to provide a conservative calculation of expected incidence rates. Transition probabilities for other event outcomes in the model were drawn from a review of the literature (see Table 1).

**Table 1:** Input Parameters

Parameter	Base Case Value	Range	Reference
Variable Input parameters			
Transition Probabilities			
Healthy to Stroke	Calculated by age and sex	–	[12]
Healthy to MI	Calculated by age and sex	–	[12]
Healthy to CHF	Calculated by age and sex	–	[13]
Healthy to Death	Varies by age and sex	–	[15]
Invariable Input parameters			
Transition Probabilities			
Acute MI to Death	0.071328306	0.057–0.086	[16]
Acute MI to Post-MI	0.928671694	–	Calculated
Post-MI to CHF	0.021759765	0.017–0.026	[16]
Post-MI to Death	0.028583536	0.023–0.034	[16]
Remain in Post-MI	0.9496567	–	Calculated
Stroke to Death	0.069	0.055–0.083	[17]
Stroke to Post-Stroke	0.931	–	Calculated
Post-Stroke to Death	0.236	0.189–0.283	[17]
Remain in Post-Stroke	0.236	–	Calculated
CHF to Death	0.43	0.344–0.516	[18]
Remain in CHF	0.57	–	Calculated
Costs ($)^a^			
Program Costs	127	102–152	Calculated
Stroke (once)	33,216	26,573-39,859	[19]
Post-Stroke (annually)	32,550	26,040-39,060	[20]
MI (once)	63,791	51,032–76-549	[21]
Post-MI (annually)	4106	3285-4927	[22]
CHF (annually)	13,619	10,895 − 16,342	[23]
Death	15,020	12,016-15,020	[21]
Utilities			
Stroke	0.64	0.512–0.768	[24]
Post-Stroke	0.66	0.528–0.792	[24]
MI	0.7	0.56–0.84	[25]
Post-MI	0.88	0.704–0.95	[26]
CHF	0.71	0.568–0.852	[27]

Costs represented in 2015 U.S. Dollars

### Model inputs: costs

Costs were calculated by summing program costs and costs associated with adverse events (see Table 1). These values were used to calculate incremental costs and benefits for analysis. Costs were calculated in 2015 U.S. Dollars, and discounted at a rate of 3%. Costs for acute events (stroke and MI) occur once, while costs for continuous event states (post-MI, post-stroke and CHF) were applied annually. For deaths associated with an acute cardiac event, a value consisting of a weighted average of costs associated with fatal MI, ischemic and hemorrhagic stroke was applied. Program costs represented a year’s cost for program operation. These costs include staff time for program directors and managers, infrastructure costs associated with hosting and maintaining the OSCAR system, and other expenses, such as travel costs, educational materials, and testing supplies. Site-specific costs were included in the total program costs. A sample budget for an individual site is provided in Table 2 and includes both site-specific costs and costs for program services that span multiple sites. The overall program costs were divided by the number of clients served in fiscal year 2015, creating a cost per-client, per-year of $126.95. This value was applied to the Colorado Heart Healthy Solutions intervention group for the first 2 years of the model, assuming the average participant would be enrolled for 2 years. To account for potential bias created by including only participants with both test and retest screening data, an intention-to-treat approach was taken. The cost per-client, per-year figure was applied twice in the first year of the model to account costs associated with participants who were screened but excluded from the analysis data set due to not having returned for follow-up or having missing values. The societal perspective was used in this analysis.

**Table 2:** Program Costs

Program Costs	
Staffing Salary	
Program Director	31,934
Medical Director	30,019
Senior Program Manager	79,334
Associate Program Manager	31,380
Infrastructure	
Maintenance of OSCAR data system	22,142
Hosting OSCAR	28,000
General Costs	
Travel	7828
Community Health Worker Training	8985
Testing Supplies	78,264
Educational Materials	19,398
Postage	922
Site Costs (Sample Budget)	
Staffing Salary	
Community Health Worker	32,854
Supervisor	5265
Walking Club Coordinator	2335
Pass-Through Costs	
Travel for trainings and screenings/retests	2598
Cell Phone	600
Office/Medical Supplies	600
Walking Club Supplies	500
Postage/Shipping	360
Promotion/Printing	400
Indirect costs	
Indirect Rate (10%)	4551
Site Total	50,063
Total Cost Per Client Per Year	126.95

### Model inputs: utilities

Quality adjusted life-years (QALYs) were calculated using utility data drawn from the literature (Table 1). QALYs are a standard measure of health used in health economics; QALYs are a continuous measure ranging from 1 (which represents full health) to 0 (which typically represents death). The “disutility value” represents the decline in health associated with the state. Total QALYs were calculated by multiplying the length of time spent in the health state by the utility value associated with each state. Future QALYs were discounted at the same discount rate as the costs.

### Analyses

In all analyses, risk factors were calculated from sample averages of participants’ initial screening values (i.e., the parameters in the model). The primary analysis included both the overall population and at-risk populations. Values from the initial screening were used for analysis of the no-intervention group. The values used to represent the treatment scenario were the final re-screening values of Colorado Heart Healthy Solutions participants following intervention. It was assumed that participants that received initial screening but did not return for follow-up received no health benefits from the screening. Secondary analysis was also performed using screening values from only the at-risk population [10]. See Table 3 for an overview of the values utilized for each scenario stratified by gender.

**Table 3:** Base Case and At-Risk Scenario Analyses

Parameter	Base Case Male		Base Case Female		At-Risk Male		At-Risk Female	
	Standard	CHHS	Standard	CHHS	Standard	CHHS	Standard	CHHS
Age	52	52	52	52	52	52	52	52
Baseline systolic BP, mm HG	131	128	125	123	134	129	131	127
Total Cholesterol	195	189	199	195	199	191	207	200
HDL cholesterol	41	44	53	55	40	44	51	53
Heart Rate	80	80	80	80	80	80	80	80
Smoke	No	No	No	No	No	No	No	No
Diabetes	No	No	No	No	No	No	No	No
CVD	No	No	No	No	No	No	No	No
LVH	No	No	No	No	No	No	No	No
Valvular Disease	No	No	No	No	No	No	No	No
Total Costs ($)^a^	30,114	26,538	21,458	19,570	33,002	27,305	27,401	16,923
Total QALYs	15.37	15.53	15.95	16.04	15.24	15.49	15.65	16.01
Incremental Cost ($)^b^	-3576		-1889		−5697		−10,478	
Incremental QALYs^c^	0.16		0.08		0.26		0.36	
ROI^d^	9.39		4.96		14.96		27.51	

Costs represented in 2015 U.S. Dollars b Incremental cost represents cost of Colorado Heart Healthy Solutions intervention minus cost of no intervention c Incremental QALYs represent QALYs associated with Colorado Heart Healthy Solutions intervention minus QALYs of no intervention d Return on investment represents net returns of the program divided by investment in program costs

In a series of one-way sensitivity analyses, the assumptions of the model were individually tested to determine if the model outputs were sensitive to any of the parameters. Transition probabilities, utilities, costs and discount values were varied one at a time. In the main analysis, the treatment effect was held constant for the length of the model. To determine the timeframe that the treatment effect must last for the program to break even, an analysis was performed in which the persistence of the treatment effect varied. In this analysis, after the treatment effect expired, the transition probabilities for adverse events in the treatment group became equal to those of the no intervention group. The year in which the treatment effect expired was varied, starting with a persistence length of 2 years (the length of program participation). The discount rate was varied from 0 to 6%. The baseline total cholesterol level was varied between 185 and 205 mg/dL. The cycle year when the incremental costs were closest to zero, while still being cost saving, was identified. This scenario analysis determined the impact the persistence of the treatment effect had on the outcome of the model. The break-even analysis calculated how long the treatment effect must persist for the program to break even for males and females.

Finally, return on investment was calculated, which was defined as the net returns from the program divided by the investment in the program [28] where the incremental costs of the model were divided by the program costs for the first 2 years.

## Results

### Base case results

In the base case of a 52-year-old male participant, individuals in the Colorado Heart Healthy Solutions intervention had lower estimated discounted total costs ($26,538) than the comparison scenario of no intervention, in which the baseline risk factors did not change ($30,114). Overall, total spending, including both increases in spending due to the program costs ($366) and reductions in spending due to averted medical care costs ($3942), were approximately $3576 less for the Colorado Heart Healthy Solutions intervention than for the comparison scenario.

Participants in the program had 15.53 QALYs, while the comparison scenario yielded 15.37 QALYs, for a gain of 0.16 QALYs. With both lower costs and a positive incremental QALY gained, the Colorado Heart Healthy Solutions strategy was dominant for males. Similarly, for the 52-year-old female base case, Colorado Heart Healthy Solutions showed discounted total costs of $19,570 and 16.04 QALYs. The comparison scenario of no intervention showed discounted costs of $21,458 and 15.95 QALYs. The incremental cost savings of Colorado Heart Healthy Solutions were $1889, with 0.08 QALYs gained.

### At-risk population results

The analysis was then estimated using a scenario of a 52-year-old male considered at-risk for developing CVD. Among at-risk participants, Colorado Heart Healthy Solutions had total discounted costs of $27,305 and 15.49 QALYs. In the comparison scenario, the resulting discounted costs were $33,002 and 15.24 QALYs yielding an incremental cost savings of $5697, and incremental effectiveness of 0.26 QALYs gained. For the at-risk 52-year-old female scenario, Colorado Heart Healthy Solutions had total discounted costs of $16,923 and 16.01 QALYs. Without intervention, the at-risk female scenario resulted in discounted costs of $27,401 and 15.65 QALYs. The incremental cost of Colorado Heart Healthy Solutions was a savings of $10,478 and the incremental effectiveness was 0.36 QALYs gained.

### One-way sensitivity and break-even analyses

All one-way sensitivity analyses continued to show Colorado Heart Healthy Solutions as dominant over the comparison scenario for all inputs. Smoking cessation, discount rate, and baseline total cholesterol level had the largest influence on the incremental cost of the intervention. For the incremental benefits, smoking cessation, discount rate, and smoking status had the largest impacts on the model. Smoking cessation created cost savings of $28,317 and created 1.26 QALYs. Varying the discount rate from 0 to 6% resulted in a range of cost savings from $6034 to $2230, and generated QALYs from .29 to .09. Varying the baseline total cholesterol level between 185 and 205 mg/dL created a range of cost savings from $1310 to $3741. None of the input variations changed the outcome from cost saving to cost spending, nor did they cause the benefits to change from creating QALYs to losing QALYs.

The break-even analysis showed that in the base case male scenario, the treatment effect must persist for 4 years for the program to break even. In the base case female scenario, the treatment effect must persist for 6 years. In the at-risk scenarios, the break-even point was 3 years for the male group and 2 years for the female group.

### Return on investment

The return on investment calculations for the base case male scenario showed an ROI of 9.39.

In the base case female scenario, the ROI was 4.96. The at-risk male scenario had an ROI of 14.96, and the at-risk female scenario showed an ROI of 27.51.

## Conclusions

Colorado Heart Healthy Solutions has been previously shown to be effective in reducing risk factors associated with global cardiovascular disease risk [10]. To our knowledge, this is the first study demonstrating that a public health program was a cost-effective method of reducing CVD risk. We found that Colorado Heart Healthy Solutions is a cost-effective strategy, which generated cost savings through averted CVD events and suggests that community-based programs may have a role improving population health beyond traditional healthcare delivery.

The models showed small gains in QALYs, but combined with the incremental cost savings of the program, the program was dominant compared with no intervention. In the base case of a 52-year-old male participant with standard risk factors, the intervention was associated with a cost savings of $3576 and a gain of 0.16 QALYs. For a female participant of the same age with average risk factors, participation was associated with a cost savings of $1889 and a gain of 0.08 QALYs. This gender difference was expected due to the lower overall cardiovascular disease risk among women; a lower starting risk translates into less overall benefit. While there is a smaller incremental cost/benefit among female participants, the program is still cost-effective. As expected, program impact and cost-effectiveness was magnified among at-risk populations. For a 52-year-old male determined to be at-risk for cardiovascular disease, the program was associated with cost savings of $5697 and generated 0.26 QALYs. For a woman of the same age who is at-risk, the intervention saved $10,478 and had an incremental benefit of 0.36 QALYs gained.

## Discussion

This study provides evidence supporting the cost-effectiveness of community health worker-based interventions. Previous studies of CHW-based interventions have provided insufficient evidence regarding cost-effectiveness, and limit comparison to other intervention types [7]. By providing incremental cost and benefit information, this study adds to the literature regarding the feasibility of implementing CHW-based interventions for reducing CVD risk.

The study has several important limitations. First, the Markov model does not include recurrent CVD events. A patient was assumed to experience a single stroke or MI, which may have led to underestimating total outcome events and cost savings of the program. We attempted to address this issue by applying age-specific calibration factors to the model to bring the number of observed outcomes closer to published incidence rates. The model may still have underestimated the number of events, as calibration factors used were conservative. This would likely minimize the observed effect, making the program potentially more effective. Second, our model held CVD risk factors constant over time. The transition probabilities were recalculated by age, but systolic blood pressure, total cholesterol and high-density lipoprotein cholesterol were held constant, even though risk factors generally worsen over time given expected temporal increases in body mass index. Our model did not account for additional prescription drug costs that a program participant might incur. We performed a scenario analysis in which a cost of $100 per year was applied to the treatment group for the life of the model, to account for additional prescription drug costs given widespread availability of generic lipid-lowering and anti-hypertensive drugs. In the base case male scenario, the program realized a cost savings of $1616, a difference of $1960 in cost savings from the primary analysis. However, the program remains cost saving, even with the prescription drug costs included. Arguably, the base case could have included the prescription drug costs, however the conclusions of the study would not change. Third, in a controlled setting such as this study, it is possible there could have been an improvement in the unobserved control arm due to secular trends. Fourth, the model used calculations based on the Framingham Heart Study instead of the newer atherosclerotic cardiovascular disease (ASCVD) Risk Estimates [29], as the OSCAR system was developed prior to this formula being published. Finally, the cost of office space was not available to the research team and is not included. Also, although this study takes a societal perspective, given the inputs and costs included in the model, the results are very similar to results from a payer perspective.

One distinguishing feature of the program is the repeated follow-ups performed by CHWs, which served to reinforce the intervention, effectuate behavior change, and have previously been shown in multi-variable analysis to be associated with greater improvements in CVD risk [10]. While data are not available on long-term persistence of the interventions effect on risk-factor control, the break-even analysis showed that the intervention effect does not need to persist very long after the intervention for the program to be cost neutral, particularly among at-risk participants.

Programs such as the one reported herein have faced several obstacles to widespread adoption. One obstacle is a lack of evidence about not just about effectiveness, but also about cost effectiveness. We show that a population-based prevention program can be cost saving from the societal perspective, with even greater savings if the program is targeted toward high risk populations. A second obstacle is a payment system that rewards volume rather than value. As the health system transitions toward value-based rewards for healthcare systems, interest in ways to promote health will become important. Evidence of the type presented here may encourage more widespread adoption of community-based prevention programs.

## Conclusions

We find that the use of community health workers to improve lifestyle and reduce CVD risk factors both increases quality adjusted life years and reduces net spending. Savings are dependent on both age and gender, with incremental cost savings of $3576 for a 52-year-old man and $1889 for a woman of the same age. This suggests that population based health programs have the potential to complement primary care preventative services and both improve health and reduce total medical care costs.

## Data Availability

The datasets used and/or analyzed during the current study are available from the corresponding author on reasonable request.

## Abbreviations

BP: Blood pressure
CHF: Congestive heart failure
CHW: Community health worker
CVD: Cardiovascular disease
HDL: High-density lipoprotein
LVH: Left ventricular hypertrophy
MI: Acute myocardial infarction
OSCAR: Outreach Screening and Referral
QALY: Quality-adjusted life years
ROI: Return on investment
